# Molecular Epidemiology of Reemergent Rabies in Yunnan Province, Southwestern China

**DOI:** 10.3201/eid2009.130440

**Published:** 2014-09

**Authors:** Hai-Lin Zhang, Yu-Zhen Zhang, Wei-Hong Yang, Xiao-Yan Tao, Hao Li, Ji-Chao Ding, Yun Feng, Du-Juan Yang, Juan Zhang, Jiang He, Xin-Xin Shen, Li-Hua Wang, Yun-Zhi Zhang, Miao Song, Qing Tang

**Affiliations:** Yunnan Institute of Endemic Diseases Control and Prevention, Dali, China (H.-L. Zhang, Y.-Z. Zhang, W.-H. Yang, J.-C. Ding, Y. Feng, D.-J. Yang, J. Zhang, J. He, Y.-Z. Zhang);; Yunnan Provincial Center of Virus and Rickettsia Research, Dali (H.-L. Zhang, Y.-Z. Zhang, W.-H. Yang, J.-C. Ding, Y. Feng, D.-J. Yang, J. Zhang, J. He, Y.-Z. Zhang);; Chinese Center for Disease Control and Prevention, Beijing, China (X.-Y. Tao, H. Li, X.-X. Shen, L.-H. Wang, Q. Tang);; Liupanshui Vocational and Technical College, Liupanshui, China (M. Song)

**Keywords:** rabies, rabies virus, viruses, molecular epidemiology, endemic disease, public health, nucleoprotein, homology, phylogenetic analysis, dogs, China

## Abstract

Yunnan Province in China borders 3 countries (Vietnam, Laos, and Myanmar) in Southeast Asia. In the 1980s, a large-scale rabies epidemic occurred in this province, which subsided by the late 1990s. However, 3 human cases of rabies in 2000 indicated reemergence of the disease in 1 county. In 2012, rabies was detected in 77 counties; 663 persons died of rabies during this new epidemic. Fifty two rabies virus strains obtained during 2008–2012 were identified and analyzed phylogenetically by sequencing the nucleoprotein gene. Of the 4 clades identified, clades YN-A and YN-C were closely related to strains from neighboring provinces, and clade YN-B was closely related to strains from Southeast Asia, but formed a distinct branch. Rabies virus diversity might be attributed to dog movements among counties, provinces, and neighboring countries. These findings suggest that Yunnan Province is a focal point for spread of rabies between Southeast Asia and China.

## Abstract 2

This province is a focal point for spread of rabies between Southeast Asia and China.

Although rabies is distributed globally, it is especially prevalent in developing countries in Asia and Africa. China has the second highest number of deaths caused by rabies, exceeded only by India (*1**–**3*). A massive rabies epidemic occurred in China in the 1980s (*4**–**6*), which subsided by the mid-1990s. However, rabies has once again become a serious public health concern in China. A sharply increasing dog population and a lack of efficient management and vaccination of dogs, especially in rural areas, has led to a dramatic increase in human rabies cases in many provinces in China (*6**–**9*). Yunnan Province in China shows the same temporal pattern of rabies outbreaks as the rest of China; rabies was first reported in this province in 1956 (*10**,**11*). However, during the present reemergence of rabies, the threat to public health has intensified because the disease-endemic area has increased (Figure 1).

**Figure 1:**
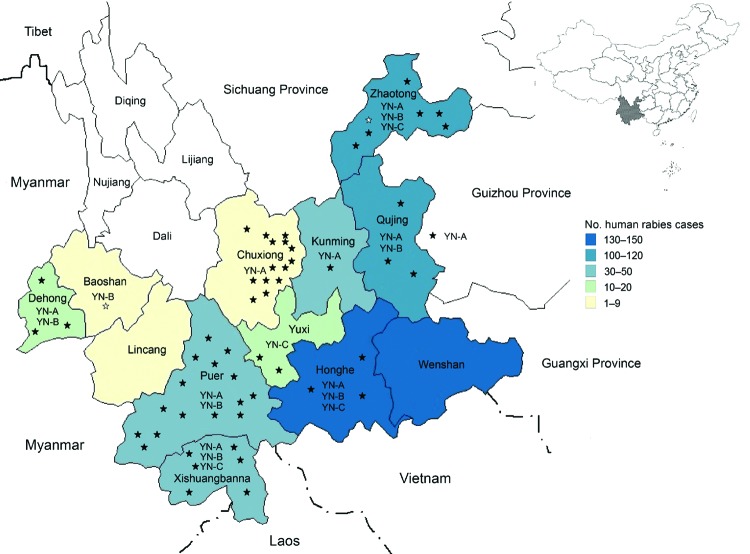
Distribution of rabies cases, 2000–2012, and clades of rabies virus isolates, 2008–2012, Yunnan Province, China. Shown are the 16 prefectures in Yunnan Province. Black stars indicate 52 specimens collected in the present study. White stars indicate specimens obtained before the present study. YN-A, YN-B, and YN-C indicate clades identified in different prefectures.

Yunnan Province, which comprises 16 prefectures and 129 counties, is located in southwestern China. It is adjacent to Guangxi, Guizhou, and Sichunag Provinces, which have the highest incidences of human rabies in China (*9*). Yunnan Province also has a 4,060 km border with Vietnam, Laos and Myanmar, which are countries to which rabies is endemic (Figure 1). This province has an area of ≈394,000 km^2^, and mountains account for >84% of the terrain; the highest elevation (6,740 m) is in the northwestern region and the lowest elevation (76.4 m) is in the southeastern region. According to 2010 census data, Yunnan Province has a population of 45.6 million persons; most persons live in the eastern part of the province. In western areas, the difference in altitude between mountain peaks and river valleys can be as much as 3,000 m.

The primary objective of this study was to clarify the epidemiology of rabies in Yunnan Province. During 2008–2012, we obtained brain tissue specimens from patients who had died of rabies and from dogs, and other animals with suspected rabies, and cerebrospinal fluid (CSF) and saliva specimens from surviving patients. Antigens of rabies viruses (RABVs) were tested, and nucleoprotein genes from 52 rabies-positive specimens were sequenced. Results were used to characterize patterns of rabies transmission and to evaluate the factors that may influence spread of rabies. Our secondary objective was to evaluate effects of rabies control and preventive measures adjusted to local conditions.

## Human Rabies Case Data

A human case of rabies was defined according to diagnostic criteria (WS281–2008) of the Health Department of the People’s Republic of China. A person with rabies had to have the following features: a history of being bitten or scratched by a dog, cat, or a wild animal, or had a wound that was licked by these animals; and clinical manifestations of itching, pain, numbness, and formication around the healed wound, followed by hyperactivity, hydrophobia, aerophobia, spasms of the pharyngeal muscle, and sympathetic excitability. The prodromal stage of paralytic rabies shows hyperpyrexia, headache, emesis, and pain at the site of the wound. Muscles of patients gradually become paralyzed, and patients with rabies die of cardiorespiratory arrest within a few days.

A case-patient with laboratory-diagnosed rabies had RABV antigen, antibody, or nucleic acid was detected in specimens. In China, rabies is a reportable disease; all human cases are reported to the Chinese Center for Disease Control and Prevention (CDC), and nearly all cases are confirmed by clinical features, rather than laboratory diagnosis. A person who satisfied these criteria was confirmed as having a clinical case of rabies.

Human rabies case data were obtained from an infectious disease database report that was officially compiled by the Yunnan Center for Disease Control and Prevention, and the Chinese CDC. Detailed information was obtained by epidemiologic investigations. These data were sorted and analyzed by using Excel (Microsoft, Redmond, WA, USA) and a descriptive epidemiologic method.

## Sample Collection

During 2008–2012, brain tissues from 1 cow and 86 sick dogs suspected of having rabies, including dogs that had bitten humans or animals (during abnormally aggressive incidents), were obtained in Baoshan, Dehong, Honghe, Xishuangbanna, Qujing, Zhaotong, Yuxi, Puer, and Chuxiong Prefectures of Yunnan Province. In addition, brain tissues from 1,069 apparently healthy dogs were collected during depopulation of dogs in 11 villages in which a rabies outbreak suddenly occurred and threatened local inhabitants. Brain tissues from 300 dogs used for meat were also obtained from local restaurants. Human brain tissues were obtained from 3 patients within 24 h of death. In addition, 14 saliva samples and 1 CSF sample were obtained from surviving patients. All specimens were kept in airtight screw-cap tubes, transported in liquid nitrogen, and stored at –70°C until tested.

## Detection of RABV Antigen

All brain tissues were analyzed by using a direct immunofluorescence assay (DFA) (*12*) and fluorescent-labeled monoclonal antibody against RABV nucleoprotein (Rabies DFA Reagent; Chemicon, Temecula, CA, USA). Fluid specimens were also screened for specific gene fragments of the nucleoprotein gene by using a nested PCR.

## Reverse Transcription PCR

Viral RNA was extracted by using TRIzol Reagent (Invitrogen, Carlsbad, CA, USA) and used as template for synthesis of cDNA with Ready-To-Go You-Prime First-Strand Beads (GE Healthcare, Piscataway, NJ, USA). Specific regions of nucleoprotein genes were amplified by using nested PCR.

## DNA Sequencing

Complete nucleoprotein gene sequences were obtained by using primers specific for this gene as described (*12**,**13*). PCR products were purified by using a QIAquick PCR Purification Kit (QIAGEN, Hilden, Germany) and sequenced.

## Phylogenetic Analysis

Complete nucleoprotein gene sequences were analyzed by using BioEdit software (http://bioedit.software.informer.com/) and ClustalX version 1.8 (http://www.clustal.org/) software. Nucleotide homologies were analyzed using the MegAlign software version 5 (DNAStar, Madison, WI, USA). Phylogenetic trees were generated by the neighbor-joining algorithm in MEGA version 5 (http://www.megasoftware.net/).

## Distribution of Human Cases

### Temporal Distribution

On the basis of published reports, during 1956–2012, a total of 1,841 human rabies cases were reported officially in Yunnan Province (annual average incidence 0.115 cases/100,000 population; range 0.00–0.69 cases/100,000 population). However, only 33 cases occurred during 1956–1979. In the 1980s, the number of human cases increased and accounted for 57.9% of the total number since official records were initiated in 1956. The highest numbers of human rabies cases were reported in the late 1980s. From 1990 onwards, the number of reported cases fell from 73 cases in 1991 to 5 cases in 1994; during 1995–1999, human deaths caused by rabies were rare. However, this decreasing trend in the incidence of rabies then reversed; 3 cases were reported in 2000, and the number of cases increased to 130 by 2010. During 2000–2012, a total of 663 human cases were reported.

### Regional Distribution

**Figure 3:**
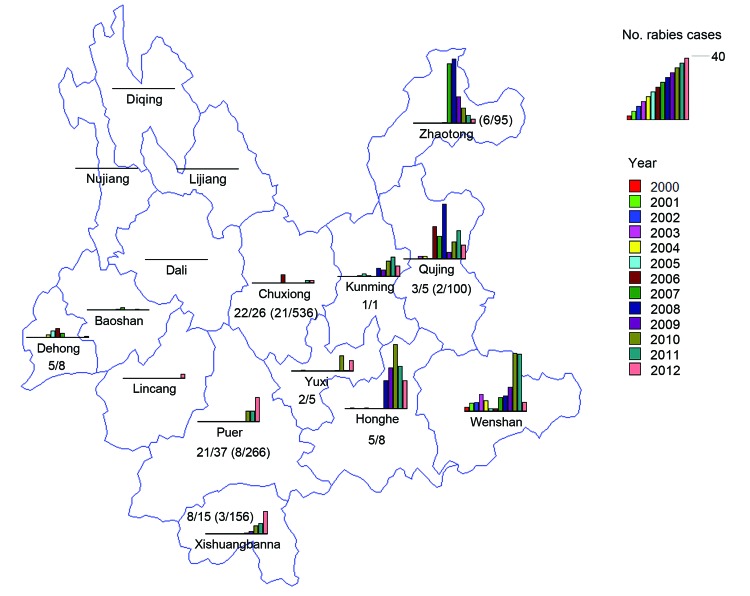
Distribution of rabies cases in 16 prefectures in Yunnan Province, China, 2000–2012, and data analysis of human and animal specimens. Except for Dali, Lijiang, Nujiang, and Diqing Prefectures, 12 prefectures had reported human cases. The color key is a scale in which each color bar indicates the year and its length indicates the number of human cases in that year. The longest bar indicates 40 human cases. Values indicate number of rabies-positive samples/number of samples submitted for testing from sick dogs, dogs with suspected rabies, and patients. Values in parentheses indicate number of rabies-positive samples/number of samples submitted for testing from apparently healthy dogs in areas to which rabies is epidemic. During 2008–2012, a total of 1,267 specimens from 9 prefectures were submitted for testing, including 95 brains of apparently healthy dogs from Zhaotong where rabies is endemic (6/95); five brains of sick dogs (3/5) and 100 brains of apparently healthy dogs (2/100) from the rabies-endemic area of Qujing; 1 human brain (positive) and 7 saliva samples of patients with rabies (4/7) from Honghe; 5 saliva samples (2/5) from Yuxi; 1 brain of a sick dog (positive) from Kunming; 26 brains of sick dogs or dogs with suspected rabies (22/26) and 536 brains of apparently healthy dogs (21/536) from rabies-endemic areas in Chuxiong; 36 brains of sick dogs or dogs with suspected rabies (20/36), 1 brain of 1 sick cow, and 266 brains of apparently dogs (8/266) from rabies-endemic areas in Puer; 12 brains of sick dogs or dogs with suspected rabies (5/12), 165 brain samples of apparently healthy dogs (3/165), 1 human cerebrospinal fluid sample (positive), and 2 saliva samples (positive) of patients with rabies from the rabies-endemic area of Xishuangbanna; 7 brains of sick dogs or dogs with suspected rabies (4/7) and 1 human brain sample (positive) from Dehong; and 1 human brain sample (positive) from Pan County in Guizhou Province, a county bordering Qujing Prefecture in Yunnan Province. Samples were not obtained from Wenshan, Lincang, Dali, Baoshan, Lijiang, Nujiang, and Diqing Prefectures.

**Figure 2:**
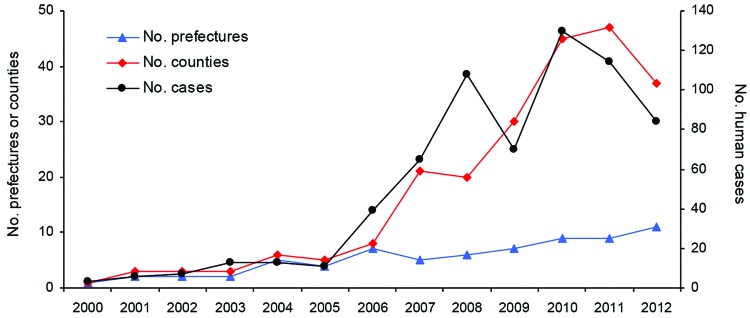
Temporal trends of rabies-affected prefectures, counties, and human cases, Yunnan Province, China, 2000–2012. Yunnan Province is divided into 16 prefectures and 129 counties.

During the past 13 years, rabies-endemic areas in Yunnan Province showed an increase in the number of rabies cases. In 2000, a total of 3 human deaths caused by rabies were reported in 1 county in Wenshan Prefecture. By the end of 2012, human cases were detected in 12 prefectures (77 counties). However, no cases have been reported in 4 prefectures in the northwestern Yunnan Province since 2000 (Figures 1–3).

In Yunnan Province, most cases occurred in eastern and central areas (Figures 1, 3). Zhaotong, Qujing, Honghe, and Wenshan were the most affected prefectures in rabies-endemic areas of Yunnan Province, which borders Guizhou, Guangxi, and Sichuang Provinces. Human cases were reported in these 3 provinces (maximum no. cases: 664 in Guizhou Province in 2006; 518 in Gaungxi Province in 2006; and 372 in Sichuang Province in 2007; Center for Public Health Surveillance and Information Service and China CDC). This proximity might have been responsible for introduction of RABV into Yunnan Province. Prefectures with a comparatively low incidence of human rabies are located in southern and southwestern regions of Yunnan Province, which borders Myanmar and Laos.

Since 2000, Wenshan Prefecture has been the most affected prefecture in Yunnan Province; a high incidence of human rabies was reported during 2007–2011 (Figure 2). Furthermore, rapid increases in numbers of human cases were reported in Qujing, Zhaotong, Honghe and Kunming Prefectures during 2006, 2007, 2008, and 2010, respectively; the number of cases in Yuxi, Puer, and Xishuangbanna Prefectures also began to show an increasing trend. This distribution pattern suggests that the disease might have spread from east to west (Figure 3). In Baoshan and Dehong Prefectures in western Yunnan Province, human rabies cases were documented every year during 2004–2007; there was a decrease in cases during 2008–2010 and an increase in cases during 2011–2012. However, in 2012, four human cases were reported in Lincang Prefecture in Yunnan Province, where no rabies cases had been reported before 2011.

### Seasonal and Demographic Distribution

Human rabies cases were reported year-round. However, reports of human cases usually peaked during May–October. Adult farmers were the most common case-patients (64%), followed by school-age children living in the countryside. Frequency of cases in male patients (73%) greatly exceeded that in female patients (27%).

## RABV Detection in Specimens

All brain tissues were examined by DFA for RABV antigen. Three human brains were confirmed to be positive for RABV. Of 86 brains from sick (rabid) dogs and dogs suspected of having rabies, 54 were positive for RABV, and 45/47 dogs that bit humans were positive for RABV. In addition, 1 bovine brain was positive for RABV. The frequency of rabies-positive dogs in rabies-endemic villages was 3.44% (40/1,162) (Figure 3), and 300 specimens obtained from dogs used as food in restaurants were negative. Eight of the 14 saliva samples and 1 CSF sample were confirmed as positive for RABV RNA.

## Nucleoprotein Gene Sequencing

Complete gene sequences of the nucleoprotein gene were obtained from 52 RABV-positive specimens: brain specimens from 42 dogs, brain specimens from 3 persons who died, 1 specimen from a cow that died, 5 human saliva specimens, and 1 human CSF specimen. All 52 sequences were submitted to GenBank under accession nos. JF819603–JF819612, JF819614-JF819624, JQ040591–JQ040600, and JX276405–JX276425.

## Molecular Diversity and Phylogenetic Analysis

**Figure 4:**
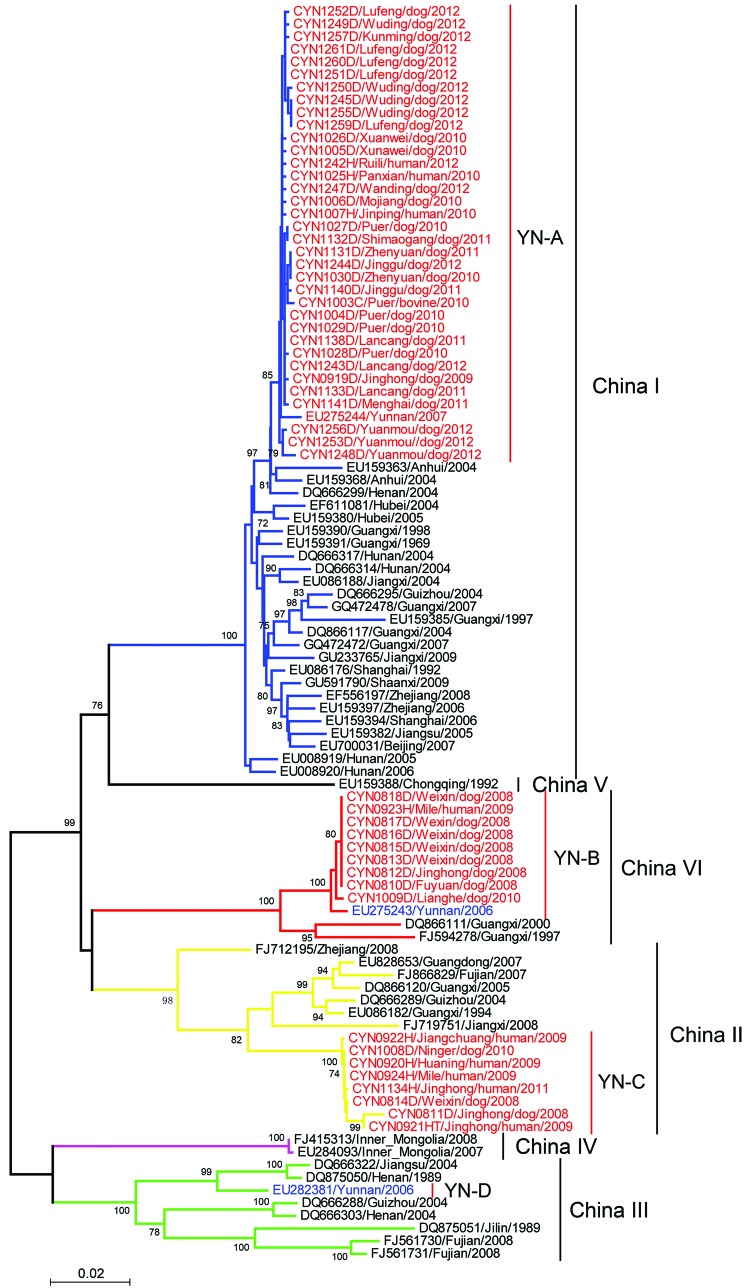
Phylogenetic relationship of nucleoprotein gene sequences of rabies virus isolates from Yunnan Province, China, 2008–2012, with isolates from neighboring provinces in China. Numbers at each node indicate degree of bootstrap support (only values >70% are indicated). Red indicates taxa sequenced in this study; blue indicates taxa from Yunnan Province; black indicates taxa from other provinces in China. Blue branches indicate China I clade; yellow branches indicate China II clade; green branches indicate China III clade; purple branch indicates China IV clade; black branches indicate China V clade; red branches indicate China IV clade. Scale bar indicates nucleotide substitutions per site.

Phylogenetic analysis showed that the 52 RABV s isolated belonged to group I, clustered in 3 clades (designated YN-A, YN-B, and YN-C) (Figure 4), and had distinct distribution characteristics (*14**–**17*). YN-A, which contained 35 viruses, circulated primarily in eastern and central Yunnan Province; 1 virus (CYN1025H) was obtained from a patient who had been exposed to RABV in Pangxian County in Guizhou Province, which is adjacent to eastern Yunnan. A virus in this clade was identified in 2012 in a patient who lived in Yunnan Province near Myanmar. Nine YN-B viruses were obtained in western and southern Yunnan Province near the border with Myanmar and in northeastern Yunnan Province. YN-C, which contained 8 viruses, was found usually in the central and southern regions of Yunnan Province.

In general, RABVs obtained from the same prefecture or county within a similar time frame clustered in the same clade, as shown by a canine rabies outbreak in Chuxiong Prefecture in 2012. However, with the spread of RABV over time, viruses in the same clade, such as YN-A and YN-B, were found in the eastern and western regions of Yunnan. This finding suggests that long-distance transmission of RABV is closely connected with the movement of dogs.

A nucleoprotein gene sequence of RABV previously isolated in Yunnan Province belongs to another clade (YN-D) (Figure 4). Thus, 4 phylogenetic clades are present in Yunnan Province YN-A, a distinct branch of the China group of RABVs (Figure 4), showed a close relationship with RABVs from most neighboring provinces. Thus, it is highly likely that these viruses had been introduced into Yunnan Province and then spread to other areas. YN-C belongs to China clade II, which circulates primarily in provinces in southern China, such as Guangdong, Guangxi, Fujian, Jiangxi, and Zhejiang (Figure 4). In Yunnan Province, YN-D (which belongs to China clade III) appeared to be present at a low prevalence because no other specimens of this type were documented.

**Figure 5:**
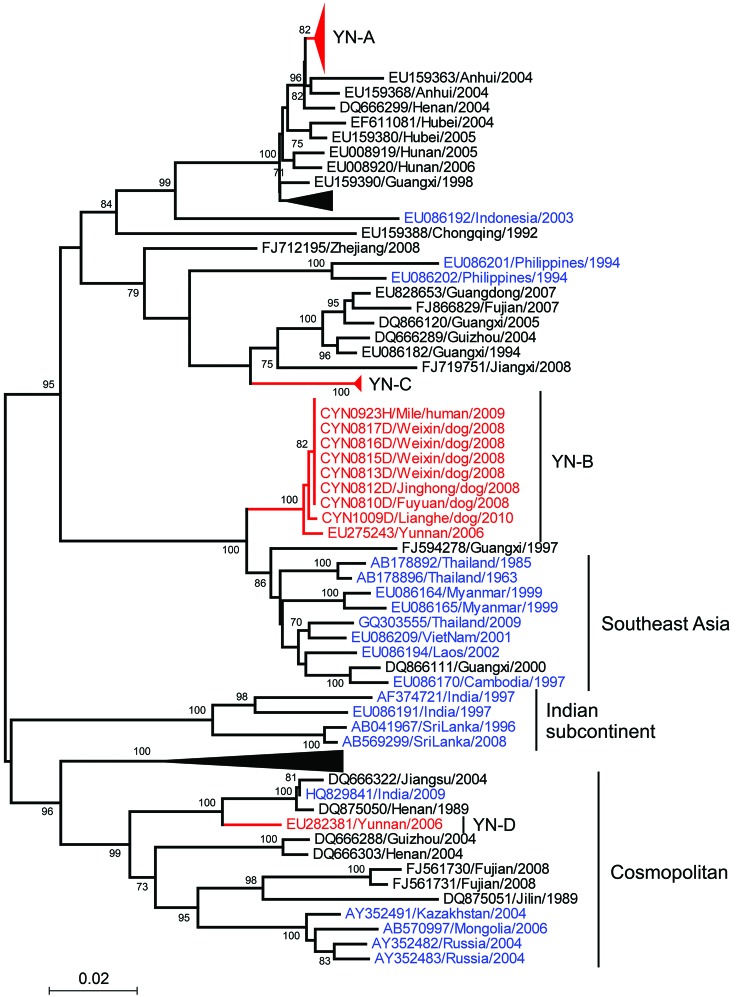
Phylogenetic relationship of nucleoprotein gene sequences of rabies virus isolates from Yunnan Province, China, 2008–2012, with those from neighboring countries in Asia. Numbers at each node indicate degree of bootstrap support (only values >70% are indicated). Red indicates taxa from Yunnan Province; blue indicates taxa from Asia; and black indicates taxa from other provinces in China. Black triangles indicate virus isolates shown in Figure 4. Scale bar indicates nucleotide substitutions per site.

YN-B is phylogenetically similar to RABVs in Thailand, Myanmar, Laos, Vietnam, and Cambodia, but formed a distinct branch within China clade IV. Results show that RABVs from Guangxi Province, which borders Vietnam, remained within the same branch as RABVs from other countries in Southeast Asia (Figures 4, 5).

## Conclusions

In China, dogs are a major reservoir of RABVs (*14*), although wildlife rabies has been reported occasionally in southeastern China (*15**,**18*). In Yunnan Province, domestic dogs are the predominant source of human rabies (*10**,**11**,**19*), and no wildlife rabies cases have been found.

Rabies was previously reported in only 1 county in Yunnan Province in 2000, but has now been reported in 77 counties in this province. Despite the obvious increase in the dog population, canine rabies has remained a neglected disease in Yunnan over recent decades. Currently, rural families in many villages commonly own 2–3 dogs. However, there is no national or provincial law or related department to ensure compulsory annual routine vaccinations in dogs. Because of this large reservoir of susceptible animals, dog-to-dog transmission of RABV has been difficult to interrupt.

Yunnan Province is affected not only by neighboring provinces, but also by other countries in Southeast Asia because of the extensive border. Two investigations aggregately documented 5 rabid dogs from Myanmar that entered villages in China (Dehong Prefecture); attacked humans, pigs, horses, and other domestic animals; and caused turmoil in the affected rural communities (*20**,**21*). In the present study, RABV from all 15 brain specimens of dogs obtained from 5 counties in Chuxiong Prefecture within a 6-month period (47 abnormally aggressive incidents) were grouped into clade YN-A. This grouping suggested a dramatic rabies outbreak in dogs associated with the traditional laissez-faire dog-keeping practice.

At least 2 mechanisms of canine translocation due to human activities that could play a major role in RABV transmission exist. First, unrestricted by law, dogs can be traded over short distances at local fairs and delivered as gifts by friends or relatives who live in neighboring villages that are not subject to a quarantine process. These dogs might be kept and valued principally as watch dogs that guard and protect the properties of their owners. Second, long-distance translocation can occur through restaurants that sell cooked dog meat. Although no specimens from dogs used as meat were positive for RABV in this study, some managers of local restaurants reported that their dogs were obtained from neighboring provinces or even Myanmar, and not all were sold for meat. Among dogs in Guizhou, Guangxi, and Hunan Provinces, some were confirmed as positive for rabies by Li et al. (*22*).

On the basis of studies conducted by Wu et al (*23*), which demonstrated that phylogenetic analyses of 1 gene of lyssavirus can show similar results as analysis of complete genomes, we sequenced and analyzed the nucleoprotein gene of RABVs. Our results were supported by molecular epidemiologic analysis of nucleoprotein gene sequences of RABVs. Nucleoprotein gene sequences are the most conserved of the 5 RABV genes and have been extensively analyzed (*24**–**28*). Zhang et al. (*29*) grouped 37 RABV strains from China into 2 phylogenetic clades. Four other studies (*16**,**17**,**30**,**31*) reported grouping of RABVs from China into 6 clades. In our study, we found 4 clades in Yunnan, which suggests genetic diversity of RABVs in this province.

Despite the paucity of data for Myanmar, Laos, and Vietnam before 2008, we elucidated the pattern of RABV spread. Clade YN-A, which was initially introduced into eastern Yunnan Province from neighboring provinces, probably spread westward to adjacent prefectures and might have spread into neighboring countries from which it returned to Yunnan Province (Figure 1). Early in 2006–2007 we verified the presence of clade YN-B in a human brain specimen from Longyang County and a domestic dog brain specimen (EU275243) from Tengchong County in Baoshan Prefecture, near Myanmar (*32**)*. This clade was also detected in other prefectures in this study. The phylogenetic relationship of RABVs in other countries in Southeast Asia and their independent branch (Figure 5) indicate that RABV was introduced from neighboring countries, became established locally as an enzootic virus, and then spread northward into eastern and other areas of Yunnan.

We cannot exclude the possibility that clade YN-B will spread further in China. In addition, more specimens should be obtained from Wenshan Prefecture, other prefectures, and neighboring countries to clarify the pattern of spread of YN-C. We speculate that rabies epidemics in Yunnan Province will remain complicated because of the unique location of this province, which also plays a major role in rabies dispersal among developing countries in Asia.

In eastern Yunnan Province, the high population densities of humans and dogs and the relatively advanced transportation network are advantageous for regional spread of RABV. In contrast, mountains and valleys in the northwestern province act as barriers to slow the spread of the virus. However, the major factor associated with RABV circulation in dogs is dog population density, which correlates with the human population density. In the most affected prefectures (Wenshan, Zhaotong, Qujing, Honghe, and Kunming), population densities were 109.25–299.58 persons/km^2^ and 10.76–19.81 dogs/km^2^. In Diqing, Nujiang, and Lijiang Prefectures, where no human or dog rabies cases have been reported, the population densities were 16.76–60.44 persons/km^2^ and 3.44–8.74 dogs/km^2^ (2012 census and 2012 dog population statistics derived from the Yunnan Animal Diseases Control Center). In contrast, the human:dog ratio (an indicator of the average number of persons who own a dog) was lower in Diqing, Nujiang, and Lijiang Prefectures (4.87–9.08) than in Wenshan, Zhaotong, Qujing, Honghe, and Kunming Prefectures (10.61–15.41).

Because the recommended preventive strategy (annual vaccination) has not been implemented in the dog population in Yunnan Province, the following temporary interventions and remedies are suggested for a village affected by rabies. First, persuade villagers to confine their dogs, regardless of any negative response. Second, euthanize unsupervised dogs and domestic dogs within the affected villages. Third, vaccinate all dogs in neighboring villages as an emergency and temporary measure. Fourth, vaccinate all susceptible persons, educate inhabitants regarding the risk of rabies, and subsidize poor persons for expenses relating to these issues.

Previous studies showed that in rabies-endemic areas, some apparently healthy dogs are infected with RABV (*22**,**33*). In the present study, of 1,162 brain samples from apparently healthy dogs in rabies-endemic areas, 40 (3.44%) were confirmed to be infected with RABV, which indicates that the virus will be disseminated by local dogs in any village containing even 1 rabid dog (Figure 3). This finding supports the rationality of culling dogs indiscriminately, given the absence of conclusive means of distinguishing apparently healthy dogs (i.e., no clinical symptoms although infected) from healthy animals. However, culling is a costly practice that usually forces local authorities into a financial dilemma. Moreover, its effectiveness is deficient because of virus transmission among dogs beyond culled areas. In contrast, vaccination of all exposed persons has been largely unrealized because of cost of rabies vaccine and human rabies immunoglobulin, as well as the ignorance of the risk of rabies in humans. Thus, human/canine rabies has continued to spread into more counties in Yunnan Province. In the near future, Dali and Lijiang Prefectures will have human cases of rabies because these areas have dog population densities of 11.02 dogs/km^2^ and 8.74 dogs/km^2^, respectively.

We did not obtain specimens from dogs in all prefectures of Yunnan Province (Figure 3). A canine rabies surveillance laboratory has not yet been established, but as described by Banyard and others (*34*), such a laboratory would play a major role in controlling spread of rabies. In addition, some regions of Yunnan Province have established canine entry–exit inspections and quarantine measures. However, Yunnan Province and other countries in Southeast Asia have extensive borders (4,060 km) with each other, and it would be difficult to restrict the movement of dogs, especially stray dogs, across these borders. Therefore, we recommend increased surveillance programs in the border areas of adjacent countries to control the spread of RABV.

The greatest obstacle to removing the threat of rabies is the low level of political commitment because dogs are not regarded as economically useful animals in Yunnan Province. After the goal of eradicating rabies in China before 2020, as proposed by the World Health Organization, was set, the principal concern undoubtedly was to stop RABV transmission among dogs (*34**–**37*). To achieve this goal, the following programs should be deployed. First, rabies vaccination of dogs should be made compulsory by passing of a national or provincial law. Second, for political and medical reasons, a database of adequate surveillance and laboratory data should be established to document dog rabies cases throughout Yunnan Province. Third, an inexpensive, safe, and highly effective dog rabies vaccine should be developed and the problematic vaccines currently used should be phased out. Fourth, a cost-efficiency analysis of rabies eradication should be performed because of political/economic considerations.
